# Orange Peel Valorization Through Citric Acid Extraction: Linking Process Conditions to Structural and Functional Properties of Pectin

**DOI:** 10.3390/molecules31122052

**Published:** 2026-06-11

**Authors:** Brian Macias-Frotto, Magdalena Rostro-Alanis, Jorge Welti-Chanes

**Affiliations:** Escuela de Ingeniería y Ciencias, Tecnológico de Monterrey, Monterrey 64849, Mexico

**Keywords:** agro-residue valorization, soluble fiber, functional ingredient, pectin extraction, orange by-products

## Abstract

Citrus processing generates large amounts of peel residues rich in pectic polysaccharides that can be valorized through eco-friendly extraction methods. This study evaluated how citric acid extraction conditions (pH 1.46–2.64, 51–96 °C, 20–70 min) influence the yield, composition, and functional properties of pectin obtained from orange peel. A central composite design (CCD) assessed the effects of pH, temperature, and time on pectin yield, degree of esterification (DE), and galacturonic acid (GalA) content. Pectin obtained under conditions maximizing each response was further characterized by structural, thermal, rheological, and functional properties. The maximum values obtained were 26.5% yield (96 °C, 70 min, pH 1.46), 84.1% GalA (74 °C, 42 min, pH 2.17), and 67.0% DE (50 °C, 70 min, pH 1.46). High yield was favored by high temperature and low pH, whereas GalA was maximized under moderate conditions, although at low recovery (5.6%). GalA-optimized pectin showed higher gel hardness (641 g) and apparent viscosity (0.64 Pa·s) than yield-optimized pectin (122 g; 0.28 Pa·s). This study demonstrates that optimizing for yield, GalA, or DE produces structurally distinct pectin fractions with different functional properties rather than a single optimal extraction condition.

## 1. Introduction

Citrus processing generates large amounts of by-products, with approximately half of each fruit’s mass discarded as peel, pulp, and seeds [1]. In 2024–2025, global orange production reached ~48 million metric tons annually, generating more than 20 million metric tons of residues [2]. This high-volume organic-rich residue poses environmental challenges but also contains valuable biopolymers, such as pectin. Pectin mainly comprises homogalacturonan domains and α-(1 → 4)-linked D-galacturonic acid residues that are partially methyl-esterified. The molecular weight of commercial citrus pectin commonly ranges between 100 and 200 × 10^3^ g mol^−1^, although variations occur with citrus variety and extraction conditions, such as pH, temperature, and severity [3,4]. It is widely used in the food industry and is increasingly incorporated into cosmetic, pharmaceutical, and medical formulations [5,6,7,8]. Overall, these factors highlight both the scale of citrus-processing waste and its potential for valorization into high-value functional materials within a circular economy framework [9,10,11].

Pectin is a structurally complex polysaccharide whose functionality depends mainly on its degree of esterification (DE) and galacturonic acid (GalA) content, which control gelling, solubility, and rheological behavior [3,12]. Conventional extraction employs mineral acids at 60–120 °C and pH 1–3, yielding ~15–30% [13,14,15]. Although effective, these methods generate acidic waste and can degrade pectin through de-esterification and chain scission [16,17]. Citric acid is a viable alternative to mineral acids due to its food-grade status, lower corrosiveness, reduced environmental impact, and easier handling and disposal, while still providing comparable pectin yields under optimized conditions [18,19]. However, its effectiveness depends on controlling extraction severity, as excessive acidity and temperature can lead to structural degradation despite improved solubilization [20].

Most studies on orange pectin extraction optimize a single response, typically yield or DE, implicitly assuming that higher recovery corresponds to improved functional performance [21,22]. As noted by [23], the value of pectin is more directly defined by techno-functional properties, such as viscosity, gel strength, and stability. Recent studies have focused on functional performance to ensure suitability for food and pharmaceutical applications [24,25].

A key limitation in related studies is the omission of GalA as a response variable, which directly reflects the purity and integrity of the homogalacturonan backbone [26]. Including GalA alongside yield and DE helps to distinguish between extraction efficiency and molecular preservation. While stronger acidity and higher temperature can improve pectin recovery, these same conditions may also promote de-esterification and chain degradation depending on extraction severity [16,22]. These opposite effects highlight a fundamental trade-off between solubilization and structural integrity. As a result, pectin obtained under yield-maximizing conditions is not necessarily functionally superior despite higher recovery.

Within this context, pH, temperature, and time define the operating conditions in which citric acid extraction balances protopectin solubilization and structural preservation [14,20,27,28]. Recent studies suggest that moderate-to-high temperatures, intermediate extraction times, and strong acidity favor pectin extraction with citric acid [14,20]. However, the relationship between extraction conditions and physicochemical and functional properties remains unclear. The present work addresses this gap by applying a central composite design (CCD) to evaluate how pH, temperature, and time affect yield, DE, and GalA. Response surface models and regression equations were used to identify conditions that separately maximize each response, generating three extraction regimes optimized for yield, GalA, or DE. Pectin recovered under these conditions was characterized by structural (FTIR, SEM), thermal (TGA), rheological, and textural analyses to link extraction severity with molecular integrity and functional performance. Rather than defining a single optimal condition, this approach evaluates how different optimization targets produce pectin fractions with distinct functional properties.

## 2. Methodology

### 2.1. Materials

Fresh Valencia oranges (*Citrus sinensis* L.) were purchased from a local supermarket in Monterrey, Mexico, washed with liquid soap and distilled water, and dried with clean towels. Ethanol (96%, *v*/*v*), acetone (≥99.5%), sodium hydroxide (1 N), phenolphthalein (indicator grade), and anhydrous citric acid (≥99.5% purity) were obtained from Desarrollo de Especialidades Químicas (Monterrey, Mexico). Galacturonic acid monohydrate (≥98%, 48280-5G-F) and carbazole (≥99%, C5132–250G) were purchased from Sigma-Aldrich (St. Louis, MO, USA). All reagents were analytical grade, and deionized water was used throughout.

### 2.2. Raw Material Characterization

The maturity index (°Brix/titratable acidity) was determined following [29]. Soluble solids (°Brix) were measured with a digital refractometer (Atago Co., Osaka, Japan), whereas, for titratable acidity (% of citric acid) of the juice, a diluted sample was titrated with a solution of sodium hydroxide (0.1 N) using phenolphthalein as an indicator. After juice extraction, peels were manually separated from the pulp and the albedo-to-flavedo ratio (*w*/*w*) was determined. Peel moisture content was evaluated according to [27] by weighing 2 g of fresh peel and drying it in an oven at 105 °C until a constant weight was reached.

### 2.3. Experimental Design

The study was divided into two parts. First, a CCD evaluated the effects of temperature, extraction time, and pH on pectin yield, GalA, and DE content using citric acid extraction. The CCD consisted of 20 runs, with factorial, axial, and six replicated center points, allowing estimation of linear, quadratic, and interaction effects. The design was centered at 75 °C, 45 min, and pH 2.05, with factor ranges of 51–96 °C, 19.8–70.2 min, and pH 1.46–2.64. These ranges cover mild-to-severe extraction conditions reported for citrus pectin recovery. Low pH values were included because acidity promotes protopectin solubilization, while the temperature and time ranges covered both conventional hot-acid extraction conditions and milder settings intended to evaluate whether relevant pectin recovery could be achieved with reduced thermal exposure and shorter processing times [14,21,27,30]. Axial points were positioned at a star distance (α ≈ 1.67) to capture curvature and ensure model rotatability.

Regression analysis and ANOVA were used to evaluate model significance and goodness of fit. The resulting second-order models were used to interpret the influence of temperature, time, and pH on each response through response surface plots and contour diagrams.

In the second stage, the fitted responses were used in Minitab^®^ (V. 22.4.0). New extraction trials were then conducted in triplicate under the predicted optimal conditions, and the resulting pectins were characterized by their physicochemical, morphological, and functional properties.

### 2.4. Citric Acid Assisted Extraction

Orange peels were dried in a single layer on a convection oven (Edel Ingenieros, Mexico) at 60 °C until constant weight. The dried material was ground using a high-speed Osterizer classic blender (Oster, Ciudad Acuña, Coahuila, Mexico) and sieved through a #40 mesh sieve (425 µm) to obtain a uniform particle size. The resulting powder was vacuum sealed in polyethylene bags and stored at 4 °C until further use.

Following a modified protocol from [27], dried orange peel powder was mixed with citric acid solution in a 1:25 g/mL ratio. Solutions with pH values from 1.46 to 2.64 were prepared with anhydrous citric acid (0.009–1.66 M). Extractions were performed in a shaking water bath (LabTech, Namyangju-si, Republic of Korea) at the designated temperature and time. After extraction, solids were removed by vacuum filtration at 50 kPa using Whatman filter paper #1 (Whatman, Little Chalfont, UK). The filtrate was cooled to 4 °C in an ice bath. Pectin was precipitated by adding cold 96% ethanol 1:1 (*v*/*v*), followed by refrigeration at 4 °C for 1 h. The precipitate was collected by sieving (100 mesh) and dried in a vacuum oven (Thermo Scientific Lab-Line, Marietta, OH, USA) at 60 °C for 18 h. The recovered material corresponded to crude pectin obtained by conventional alcohol precipitation. Pectin yield was calculated using Equation (1):

(1) $$\mathrm{Pectin}\;\mathrm{yield}\;(\%)\;=\;\frac{\mathrm{Dry}\;\mathrm{pectin}\;\mathrm{weight}\;\left(g\right)}{\mathrm{Dry}\;\mathrm{peel}\;\mathrm{powder}\;\mathrm{weight}\;\left(g\right)}\;\times \;100$$

### 2.5. Physicochemical, Structural, and Functional Characterization of Pectin

#### 2.5.1. Galacturonic Acid Content

GalA content was determined following a modified method [31]. In brief, 200 µL of a 1% (*w*/*w*) pectin solution was hydrolyzed with 3 mL of 97% sulfuric acid. Then, 100 µL of 1% (*w*/*v*) carbazole in ethanol was added to the hydrolyzed mixture, which was kept at 60 °C for 60 min to allow color development. Absorbance was recorded at 530 nm using a microplate reader (Synergy HT, BioTek Instruments, Bad Friedrichshall, Germany). GalA concentration was calculated from a calibration curve prepared with standard GalA solutions. GalA concentration was calculated from a calibration curve prepared using GalA standards (0–250 µg/mL), with blank correction applied before sample quantification. Standards and samples were analyzed in triplicate, and calibration linearity was verified (R^2^ = 0.997) before calculating sample concentrations. Results are expressed as the percentage of GalA relative to the dry weight of the pectin.

#### 2.5.2. Fourier-Transform Infrared (FTIR) Spectroscopy and DE

FTIR spectra were obtained following the procedure from [32] with minor adjustments. Approximately 5 mg of pectin powder was analyzed using a PerkinElmer Frontier FTIR spectrometer (PerkinElmer, Waltham, MA, USA) equipped with an ATR accessory. Spectra were recorded from 4000 to 400 cm^−1^ at 4 cm^−1^ resolution using 48 scans after background correction. Characteristic bands for ester carbonyl groups (~1740 cm^−1^), asymmetric carboxylate stretching (~1600–1630 cm^−1^), and hydroxyl groups (~3400 cm^−1^) were identified.

DE (%) was estimated from the ATR-FTIR spectra using the carbonyl-to-carboxylate peak ratio method. Peak heights were measured from the baseline-corrected spectra to the local maximum at ~1740 cm^−1^ (A_1740_) and ~1600 cm^−1^ (A_1600_) (Equation (2)). This approach, which is widely used for pectin characterization, was applied consistently across all samples to evaluate relative differences in esterification associated with the extraction conditions. Accordingly, DE values were interpreted as comparative indicators of esterification behavior among treatments.

(2) $$\mathrm{DE}\;(\%)=\frac{A_{1740}}{A_{1740}+A_{1600}}\;\times \;100$$

#### 2.5.3. Thermogravimetric Analysis

Thermogravimetric analysis (TGA) was performed to assess the thermal stability and degradation stages of the extracted pectin, following the procedure described by [22]. Approximately 6 mg of each dried pectin sample was weighed into ceramic crucibles and analyzed in a PerkinElmer TGA 8000 (Waltham, MA, USA). Samples were heated from 30 °C to 600 °C at 10 °C min^−1^ under nitrogen flow (40 mL min^−1^) to evaluate pyrolytic behavior, then the atmosphere was switched to oxygen and heated up to 800 °C to oxidize residual material. Data were processed using Pyris™ software (PerkinElmer, version 10.6.0).

#### 2.5.4. Scanning Electron Microscopy (SEM) Analysis

Surface morphology was examined following the method of [33]. Pectin samples were mounted on aluminum SEM stubs using double-sided adhesive tape to minimize sample disturbance. The mounted samples were sputter-coated with a gold layer ~10 nm thick using a Quorum 150T sputter coater (Quorum Technologies, Laughton, UK) to improve conductivity. Imaging was performed using a Zeiss EVO MA25 scanning electron microscope (Zeiss, Oberkochen, Germany) at an accelerating voltage of 10 kV and a magnification of 1000×.

#### 2.5.5. Texture Analysis

Following the protocol of [34] with modifications, pectin gels were prepared in cylindrical glass vials measuring 25 mm in diameter and 20 mm in height. A solution of 0.5% (*w*/*w*) pectin and 40% (*w*/*w*) sucrose was dissolved in citric acid-acidified water adjusted to pH 2., heated to 80 °C, and vortexed until complete dissolution. The solution was then stored at 4 °C for 24 h to allow gel formation.

Gel strength was measured using a TA-XT Plus Texture Analyzer (Stable Micro Systems, Robbinsville, NJ, USA) following a method adapted from [35]. A cylindrical probe (P/0.5, 15 mm diameter) was employed to compress the gels to a strain of 30% at a rate of 1 mm/s with a trigger force of 5 g. Texture profile analysis (TPA) calculated parameters included hardness (peak force during the first compression); adhesiveness (negative area in the first cycle); cohesiveness (ratio of the second to the first compression cycle); springiness (displacement ratio between cycles); resilience (ratio of the negative to the positive area in the first cycle); gumminess (hardness × cohesiveness); and chewiness (hardness × cohesiveness × springiness).

#### 2.5.6. Rheological Properties

Rheological measurements were performed using a Physica MCR 101 rheometer (Anton Paar, Ostfildern, Germany) equipped with a 25 mm crosshatched parallel-plate geometry (PP25/S) and a 1.00 mm gap. Pectin solutions at 2.0% (*w*/*w*) were prepared by dispersing the powder in deionized water under magnetic stirring for 15 min, followed by hydration at 4 °C for 12 h. Before analysis, samples were equilibrated at 25 °C, degassed under vacuum (−20 inHg), loaded onto the lower plate, and trimmed after reaching a normal force of 0.00 ± 0.02 N. Steady-shear flow curves were collected at 25 °C, over a shear rate range of 0.1 to 200 s^−1^ using a logarithmic ramp.

Flow behavior was described using the Power Law model (τ = *K*γ̇*^n^*), where τ is shear stress (Pa), γ̇ is shear rate (s^−1^), *K* is the consistency index (Pa·s^n^), and *n* is the flow behavior index. Model parameters were obtained from the linearized form of the model, log(τ) = log(*K*) + *n*log(γ̇), by plotting log(τ) versus log(γ̇), yielding a linear relationship in which the slope corresponds to the flow behavior index (*n*) and the intercept corresponds to log *K*.

#### 2.5.7. Solubility

Solubility of the pectin samples was determined using a modified [36] protocol. Approximately 80 mg of dried pectin powder was weighed into pre-dried, low-retention 2 mL Eppendorf tubes. Then, 1 g of distilled water was added, and the mixture was vortexed (Scientific Industries, New York, NY, USA) and centrifuged in a Sorvall, ST Plus Series (Thermo Fisher Scientific, Osterode am Harz, Germany) at 9500× *g* for 10 min. The supernatant was removed and the remaining pellet dried at 60 °C for 24 h to constant weight. Solubility was calculated as the percentage difference between the initial sample mass and the dried residue.

#### 2.5.8. Emulsifying Properties

Emulsifying capacity (EC) expressed as interfacial area per unit mass of pectin, was determined following [37] with minor adjustments. A total of 0.5% (*w*/*w*) pectin aqueous solution (5 mL) was mixed with 5 mL of canola oil and homogenized at 15,000 rpm for 3 min using an Ultra-Turrax at 25 °C. The fresh emulsion was diluted tenfold (1:10) with distilled water, and a 100 µL aliquot was immediately transferred to a 96-well plate. Absorbance at 500 nm was recorded at t = 0 (A_0_) using a microplate reader (Synergy HT, BioTek, Santa Clara, CA, USA). EC was calculated from the initial turbidity according to:

(3) $$EC=[(2\times 2.03\times (A_{0})\times DF)/(C\times \varphi \times \theta plate\times 1000)]$$

where EC is expressed in m^2^ g^−1^; C is the pectin concentration in the final emulsion (g/mL); φ is the oil volume fraction in the emulsion; θ_plate_ is the optical path length (calculated as sample volume (cm^3^) divided by the cross-sectional area of the well (cm^2^), in this case 0.292 cm; DF is the dilution factor; and A_0_ is the blank-corrected sample absorbance at t = 0 min. The constants 2 and 2.03 relate to optical scattering geometry and turbidity-to-surface-area conversion, as defined by [37].

Emulsion stability was evaluated by measuring the absorbance again at t = 10 min under the same conditions. Stability was expressed as the percentage of turbidity retained after ten minutes:

ES = [(A_10_)/(A_0_)] × 100 (4)

where A_10_ is the blank-corrected absorbances of sample at t = 10 min.

#### 2.5.9. Color

Color measurements were performed following [38] using a spectrophotometer (CM-5, Konica Minolta, Tokyo, Japan) equipped with a pulsed xenon lamp. Spectral reflectance was recorded from 360 to 740 nm at 10 nm intervals. Measurements employed a 30 mm diameter measurement spot (Medium Area View, MAV) to ensure representative sampling of the sample surface. The spectrophotometer was calibrated before each series using its internal white standard, guided by the CM-S100W SpectraMagicTM NX software (Version 10.6). Color readings were obtained under the D65 illuminant with specular component excluded and a 10° observer angle. Color was expressed in CIE L* a* b* coordinates, quantifying lightness (L*), red–green (a*), and yellow–blue (b*) attributes.

#### 2.5.10. Statistical Analysis

Unless otherwise stated, all quantitative analytical measurements were performed in triplicate, and results are reported as mean ± standard deviation. Functional property data were analyzed by one-way analysis of variance (ANOVA) followed by Tukey’s multiple comparison test at a significance level of *p* < 0.05. Statistical analyses were performed using Minitab^®^ 22 software (Minitab LLC, State College, PA, USA).

## 3. Results and Discussion

### 3.1. Orange Characterization

Orange juice contained 9.54 ± 0.38 °Brix and 1.36 ± 0.14% titratable acidity, corresponding to a maturity index (°Brix/acidity) of 6.94 ± 1.00. This is consistent with mid-season maturity, as characterized by balanced sugar and acid levels (maturity index 6–10) [39]. The peel fraction, with a moisture content of 79.6 ± 0.86%, represented 19.06 ± 1.45% of total fruit mass, and the albedo:flavedo weight ratio was 1.30 ± 0.19, giving an albedo share of 56.27 ± 3.52% of the total peel weight. These values show that approximately one-fifth of the fruit mass is peel and dominated by albedo tissue as the primary pectin source. The compositional profile falls within the range reported for commercial orange cultivars, where peel typically represents 18–36% of fruit weight and albedo accounts for more than half of the peel mass [29]. The high albedo proportion observed is relevant for pectin recovery, as albedo has higher homogalacturonan content and lower pigment concentration than flavedo, favoring higher extractable yield and GalA percentages [35]. Differences in maturity stage and peel tissue distribution may therefore influence pectin yield and composition by altering the proportion of extractable protopectin and the structural characteristics of the cell wall matrix [40].

### 3.2. Effect of the Variables of the Extraction Process on the Yield, GalA and DE of the Pectin Obtained

ANOVA, lack-of-fit testing, and residual analysis confirmed that the fitted equations were adequate for describing the influence of temperature, time, and pH within the tested range on pectin yield, GalA, and DE. Table 1 summarizes the extraction conditions and the corresponding experimental and predicted values obtained from the fitted quadratic models. The CCD generated a broad range of responses, with pectin yield varying from 3.4 to 20.3%, GalA from 57.3 to 77.3%, and DE from 50.9 to 67.5%. These ranges suggest that the chosen factor levels were adequate to capture the variability needed to characterize the extraction system. Repeated center point experiments showed moderate variability, particularly for GalA content, likely reflecting inherent experimental and analytical variation associated with biological raw materials and multistep extraction procedures. Predicted and experimental values closely agreed across the design space, indicating that the models captured the main trends in yield, GalA, and DE. Residuals were small and randomly distributed (S = 0.74 for yield), and no significant lack of fit was detected for any response (*p* > 0.05). The non-significant lack-of-fit results confirmed that this variability remained within the acceptable limits of the fitted response surface models. These statistical indicators provided the basis for examining which factors contributed most strongly to each response.

### 3.3. ANOVA and Regression Equations

ANOVA (Table 2) showed that the response surface models were statistically significant for describing the relationships between extraction variables and pectin yield, GalA, and DE within the experimental domain. The yield model explained 98.71% of the total variability (adjusted R^2^ = 97.56%), indicating agreement with the experimental data. For GalA, the model accounted for 71.79% of the variation (adjusted R^2^ = 64.27%), with significant effects of pH (*p* = 0.014) and the quadratic terms of time (*p* = 0.003) and pH (*p* = 0.001), while the lack of fit was not significant (*p* = 0.687). The DE model explained 79.92% of the variability (adjusted R^2^ = 74.56%), with pH as the dominant factor (*p* < 0.001) and a significant temperature–pH interaction (*p* = 0.006), whereas extraction time was not significant. The absence of significant lack of fit for all responses confirmed that the regression equations in uncoded units (Table 3) were suitable for response surfaces analysis and interpretation of the effects of temperature, time, and pH.

### 3.4. Effect of Extraction Process Conditions on Yield, GalA, and DE

**Figure 1:**
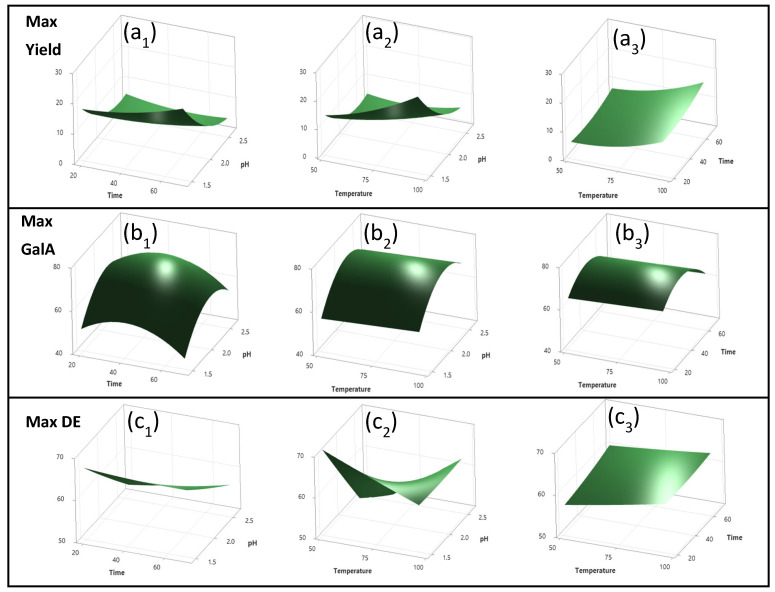
Response surface plots of the effects of temperature (°C), time (min), and pH on pectin yield (**a_1_**–**a_3_**), GalA (%) (**b_1_**–**b_3_**), and DE (%) (**c_1_**–**c_3_**) during citric acid extraction of orange peel. Each surface was generated from the fitted quadratic model, with the third variable held constant at its central value (75 °C, 45 min, pH 2.05).

The response surface plots generated from the fitted quadratic models are shown in Figure 1. These plots illustrate the combined effects of temperature, extraction time, and pH on yield, GalA, and DE, and support the interpretation of the significant linear, quadratic, and interaction effects identified by ANOVA. The following subsections describe the patterns observed in each set of plots—pectin yield (a_1_–a_3_), GalA (b_1_–b_3_), and DE (c_1_–c_3_)—and relate them to the underlying extraction mechanisms.

### 3.5. Effect on Pectin Yield

Pectin yield was mainly influenced by pH, temperature, and their interaction, consistent with the high ANOVA F-values. The second-highest experimental yield (18.65%) occurred at the lowest pH tested (1.46). Combining temperature with low pH further increased yield, reaching a predicted maximum of 20.28% at 87 °C and pH 1.70, reflecting increased dissolution of the cell wall matrix. Extraction time contributed primarily through its interaction with pH, indicating that extended time favored yield only when temperature and acidity were higher.

Response surface plots (Figure 1) show the predicted yield increased sharply when lowering pH at fixed extraction time. At 70 min (Figure 1(a_1_)), the model expects an increase in yield from about 3% at pH 2.6 to about 23% at pH 1.5. This effect increased at higher temperatures (Figure 1(a_2_)), where at 90 °C, the predicted yield increased from approximately 5% at pH 2.6 to nearly 27% at pH 1.5. The temperature–time surface (Figure 1(a_3_)) indicates that yield is predicted to increase from 3% to 15% when extraction time is extended from 20 to 70 min at elevated temperatures (85–95 °C), while longer extraction at lower temperatures provides limited benefit. These model-predicted trends are consistent with the experimentally observed yield range (3–20%). Similar yield enhancements under strong acidic and thermal conditions have been reported in acid-based extraction systems, including pectin yields up to 25.8% from ponkan peel using nitric acid and approximately 14% from garlic peels using hydrochloric acid [3,33].

### 3.6. Effect on Galacturonic Acid Content

GalA content (Figure 1(b_1_–b_3_)) displayed a complex curved response. ANOVA indicated that pH was the only significant linear term, while the quadratic components of pH and extraction time dominated the model. Response surface plots (Figure 1(b_1_–b_3_)) indicate that GalA reached a predicted maximum at pH 2 of around 75% and an intermediate extraction time (40–50 min). Across the investigated temperature range (51–96 °C), the predicted variation in GalA was limited to approximately differences of 2%, indicating a weak dependence on temperature relative to pH and time. Under more severe conditions, GalA decreased toward 40–45% at lower pH values (<1.6) or prolonged extraction times (>60 min), as reflected in the descending regions of the response surfaces.

Although the GalA model showed lower predictive strength than the yield model (R^2^ = 0.7179; adjusted R^2^ = 0.6427), it remained statistically significant with no lack of fit. The comparatively lower fit likely reflects the greater sensitivity of GalA preservation to subtle variations in extraction severity and raw material heterogeneity, which may affect homogalacturonan degradation beyond the primary effects captured by the model.

This predicted behavior is consistent with quantitative trends reported in fruit-derived pectin systems. [16] reported GalA content ranging from 66 to 81% for passion fruit peel pectin extracted at pH 2.0, with the highest GalA values obtained at lower temperatures (50–60 °C) and extraction times of 15–20 min, while increasing temperature to 90–100 °C reduced GalA to below 66% due to acid–thermal degradation of the homogalacturonan backbone. Similarly, [41] observed GalA contents above 60% under mild thermal conditions (pH 3, 58 °C), reinforcing the sensitivity of uronic acid preservation to extraction severity.

### 3.7. Effect on Degree of Esterification

DE was primarily controlled by pH, as shown by its dominant ANOVA F-value, with a significant temperature–pH interaction. The response surface plots derived from the fitted model (Figure 1(c_1_–c_3_)) demonstrate that at low temperatures (≈50 °C), decreasing pH from 2.6 to 1.46 increased DE from 45% to 70%. However, when low pH was combined with elevated temperatures (85–95 °C), the model predicted a reduction in DE to values below 55%, indicating intensified de-esterification under combined acidic and thermal stress. This interaction explains the curved DE surfaces, where high acidity increases DE only under moderate thermal conditions.

Extraction time had a minor effect on DE, appearing only through interaction terms. Predicted DE values varied by less than five percentage points across the full-time range (20–70 min) at constant pH and temperature. Overall, the response surfaces indicate that DE remained within a relatively narrow predicted range (45–72%) across the experimental domain, with pH and its interaction with temperature being the primary determinants. These trends are consistent with previous reports showing that moderate thermal conditions limit acid-catalyzed ester hydrolysis, whereas elevated temperatures accelerate de-esterification in acidic media [16,42].

### 3.8. Extraction Conditions Corresponding to the Maximum of Each Individual Variable Evaluated

Validation experiments conducted at the predicted maxima revealed trade-offs between pectin yield, GalA, and DE, as well as differences between predicted and experimental values (Table 4).

For yield optimization (96 °C, 70 min, pH 1.46), the highest experimental yield (26.5 ± 1.1%) was 5.95% lower than the model-estimated value (32.45%). A similar overestimation occurred under DE-optimized conditions (50 °C, 70 min, pH 1.46), where the experimental yield (16.7 ± 0.2%) was lower than predicted (18.44%). In contrast, yield at GalA-optimized conditions (74 °C, 42 min, pH 2.17), matched well between experimental and model (5.6 ± 0.3% vs. 5.79%). This behavior is consistent with similar studies, such as [3], who reported overestimation of pectin yield (25.6 vs. 41.0%) at the projected optimum (100 min in boiling aqueous HNO_3_ at pH 1.6) despite a strong model fit, attributing the discrepancy to degradation and reduced recoverability under high acidic conditions.

For GalA, experimental values exceeded predictions across all validation conditions. At yield-optimized conditions, GalA increased from a predicted 46.99% to an experimental 67.1 ± 6.6%. At GalA-optimized conditions, GalA increased from 72.62% to 84.1 ± 3.6%, while at DE-optimized conditions, it increased from 45.01% to 65.0 ± 4.2%. These consistent positive deviations indicate that, although the fitted model correctly identified extraction regions associated with elevated GalA, it underestimated the absolute GalA content. This suggests that GalA retention is influenced by factors not fully described by the three-variable design, reflecting the sensitivity compared to yield.

For DE, agreement between projected and experimental values was condition-dependent but remained within a narrower range than that observed for GalA. DE at the yield-optimized conditions were 62.54% (predicted) and 63.4 ± 1.38% (experimental). At the GalA-optimized conditions, DE increased from 58.7% (predicted) to 62.8 ± 1.7% (experimental), whereas at DE-optimized conditions, the experimental DE was lower than predicted (67.02 ± 0.97% vs. 72.04%). These results indicate that the validation experiments identified extraction regions associated with elevated DE, while also suggesting that DE, similarly to GalA, is influenced not only by the selected optimization target but also by extraction severity, particularly temperature and acidity.

### 3.9. Physicochemical, Structural, and Functional Characterization of Pectin Extracted at Conditions Maximizing Yield, Galacturonic Acid Content, and Degree of Esterification

Pectin obtained under the predicted conditions that maximized yield, GalA, and DE were further characterized to evaluate how each extraction target influenced their physicochemical, structural, and functional properties. This comparative analysis evaluated whether higher GalA or DE produced distinct structural or functional properties, and to assess whether extraction strategies focused on maximizing yield alone resulted in materials with comparable or inferior performance. The results highlight trade-offs between extraction efficiency and pectin quality, supporting optimization strategies beyond yield maximization alone.

### 3.10. Fourier Transform Infrared Spectroscopy (FTIR)

**Figure 2:**
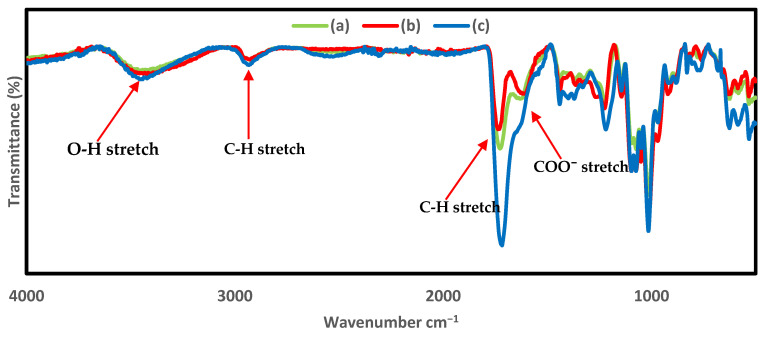
FTIR spectra of pectin obtained by citric acid extraction under conditions maximizing (**a**) yield (at 96 °C, 70 min, pH 1.46); (**b**) GalA (at 74 °C, 42 min, pH 2.17); and (**c**) DE (at 50 °C, 70 min, pH 1.46).

FTIR spectra of the three pectin samples (Figure 2) displayed the expected pectic polysaccharide profiles. All samples exhibited the broad O–H stretching band near 3400 cm^−1^ and the C–H stretching peak at approximately 2930 cm^−1^, features commonly reported in citrus pectin ATR spectra [36]. The ester carbonyl band (C=O) at ~1740 cm^−1^ and the asymmetric COO^−^ band at ~1600–1630 cm^−1^ were present in all extracts and used for the FTIR-based estimation of DE.

The relative intensities of the carbonyl bands matched well with the FTIR-derived DE values, which were 67.0% for max DE, 63.4% for max yield, and 62.8% for max GalA. Max DE displayed the most pronounced ester carbonyl peak at ~1740 cm^−1^, consistent with its higher DE. Max yield and max GalA showed similar intensities in this region, reflecting their nearly identical DE values. Although slight increases in the ~1600 cm^−1^ band were observed under the harsher extraction condition (max yield), the differences remained minor and consistent with small DE variations.

In the fingerprint region (1200–950 cm^−1^), bands at ~1145 cm^−1^ (C–O–C of glycosidic linkages) and ~1045 cm^−1^ (C–O stretching of neutral sugars) appeared sharper in max GalA and max DE, consistent with more preserved homogalacturonan structures. Similar assignments and backbone-related variations in this region have been reported for pectin systems [43].

Overall, FTIR confirmed that all extracts kept the fundamental structure of high-methoxyl pectin, with subtle variations associated with minor differences in esterification and backbone definition, which is in agreement with FTIR-based DE values [44,45].

### 3.11. Thermal Properties

**Figure 3:**
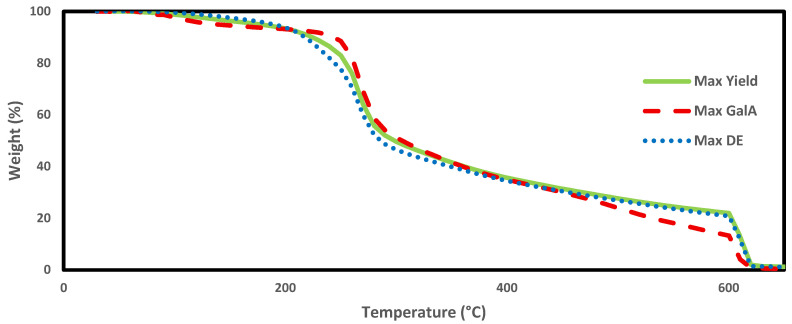
Thermogravimetric curves of pectin extracted under conditions maximizing yield (96 °C, 70 min, pH 1.46), GalA (74 °C, 42 min, pH 2.17), and DE (50 °C, 70 min, pH 1.46) showing three characteristic stages: initial dehydration (<220 °C), main depolymerization (≈220–300 °C), and oxidative burnout (>600 °C).

The thermogravimetric curves of the three pectin samples (Figure 3) showed the characteristic multi-step degradation pattern expected for citrus pectin. The first mass loss occurred below 220 °C and reflected dehydration and volatilization of physically bound water, with all samples presenting comparable mass loss (8–10%), indicating similar moisture and volatile contents. In the main decomposition region between 220 and 300 °C, all samples exhibited similar mass losses (47–50%), showing extensive depolymerization of the polysaccharide matrix.

At higher temperatures (>300 °C), differences among the samples became apparent. In this region, the curves exhibited progressive cleavage of the α-(1 → 4) glycosidic linkages within the homogalacturonan backbone and accompanying decarboxylation reactions, as commonly described for thermally degraded pectin [46,47]. Pectin extracted under GalA-optimized conditions decomposed more gradually and yielded a lower residual char (~13% at 600 °C) compared with pectin extracted under yield- and DE-optimized conditions (20–22%), indicating a reduced tendency toward char residue formation. Upon switching to an oxidative atmosphere, the remaining mass between 600 and 800 °C was attributed to combustion of carbon-based residues, indicating that differences in residue at 600 °C primarily reflected variations in char formation rather than inorganic ash content.

These findings align with reports on pectin extracted under mild and severe conditions, which also show slower decomposition and reduced char formation when the polysaccharide backbone is better preserved [43,48]. Overall, TGA indicates that differences among the samples mainly appear during the high-temperature degradation stage, with GalA-optimized pectin exhibiting lower char formation, while the initial and main decomposition behaviors remain comparable across all extraction strategies.

### 3.12. Scanning Electron Microscopy Analysis

**Figure 4:**
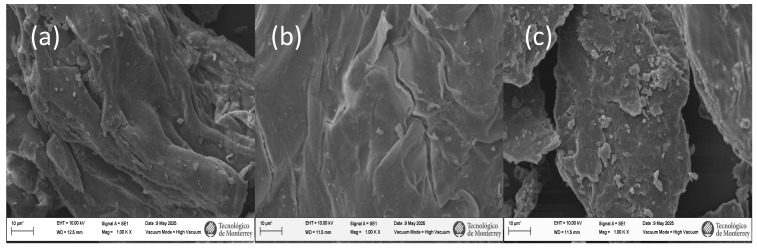
Scanning electron micrographs (1000×, 10 kV) of pectin obtained under conditions maximizing (**a**) yield (96 °C, 70 min, pH 1.46); (**b**) GalA (74 °C, 42 min, pH 2.17); and (**c**) DE (50 °C, 70 min, pH 1.46).

Microscopic analysis of pectin powders (Figure 4) revealed microstructural differences among extraction regimes. Pectin obtained under yield-optimized conditions (Figure 4a) displayed irregular, compact flakes with fused surfaces and attached particles, consistent with extraction conditions that can promote particle aggregation and denser surface features, as reported for pectin whose morphology varies with extraction chemistry and thermal conditions [32,49,50]. In contrast, GalA-optimized pectin (Figure 4b) exhibited smoother, well-defined lamellar sheets with mild folding and open inter-lamellar spaces, implying less compact structural rearrangement under moderate extraction conditions. In contrast, pectin obtained under DE-optimized conditions (Figure 4c) showed granular, cohesive particles with surface irregularities and microcracks, consistent with aggregation of more highly esterified chains during precipitation and drying.

Similar textural distinctions have been reported for citric acid and microwave-assisted extraction systems, where harsher conditions produce compact and fragmented morphologies, while moderate treatments yield smoother and more homogeneous structures [51]. Citric acid extraction has been shown to generate denser and less fractured surfaces than mineral acid- or phosphate-based systems, attributed to milder hydrolytic effects and greater retention of ester linkages [50]. Comparable layered and fragmented morphologies have also been described for pectin obtained by enzymatic, ultrasound, and microwave-assisted extraction, where extraction severity influenced particle organization and surface uniformity [38]. Overall, SEM analysis indicates that extraction severity governs pectin microstructure, with severe conditions producing compact and fused-looking morphologies, whereas intermediate conditions keep layered architectures that are consistent with the increased solubility and functional properties observed for the GalA-optimized sample.

### 3.13. Textural Properties of Pectin Gels

Texture profile analysis displayed differences in gel strength between the three pectin hydrogels (Table 5). All samples successfully formed stable, solid-state hydrogels, but these varied significantly. Max GalA formed the firmest gels (hardness of 641.3 ± 46.40 g), while max yield produced the softest (121.82 ± 16.53 g) and max DE occupied an intermediate position (336.84 ± 3.01). This pattern agrees with the role of GalA in promoting tighter junction zones and denser network formation in high homogalacturonan systems [35]. However, the max DE sample exhibited higher gel hardness than max yield, indicating that gel strength cannot be explained by GalA content alone. These results highlight the additional influence of esterification level and reduced thermal degradation, which together promote improved network integrity under less severe extraction conditions.

Statistical analysis showed that hardness and gumminess/chewiness differed significantly among the samples (*p* < 0.05), though cohesiveness, adhesiveness, springiness, and resilience remained statistically similar (*p* > 0.05). These indicates that the main contrast among the extracts lies in gel firmness, with deformation and recovery behavior remaining broadly similar—a pattern also reported for high-methoxyl gels, where compositional differences primarily affect firmness rather than elastic recovery [12].

### 3.14. Rheological Analysis

**Figure 5:**
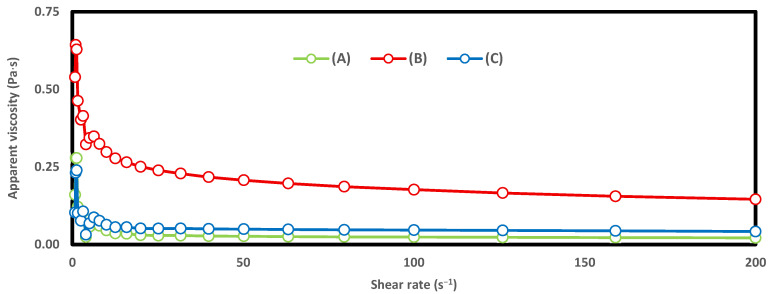
Rheological behavior of 2% (*w*/*v*) pectin solutions obtained under extraction conditions maximizing (**A**) yield (96 °C, 70 min, pH 1.46); (**B**) GalA content (74 °C, 42 min, pH 2.17); and (**C**) DE (50 °C, 70 min, pH 1.46).

The apparent viscosity of 2.0% (*w*/*w*) pectin solutions as a function of shear rate is shown in Figure 5. All samples exhibited a strong decrease in viscosity with increasing shear rate (1 to 200 s^−1^), consistent with the shear-thinning behavior usually reported for pectin solutions at comparable concentrations [48,49,52]. Contrasts in flow resistance were observed among the samples, with GalA-optimized pectin displaying the highest apparent viscosity across the entire shear rate range, followed by DE-optimized and yield-optimized pectin.

At 1 s^−1^, apparent viscosity of the GalA-optimized pectin was ~0.64 Pa·s, compared with 0.28 Pa·s for the yield-optimized sample and 0.23 Pa·s for the DE-optimized sample. These values fall within the range reported for 2% citrus pectin solutions, where low-shear viscosities between 0.2 and 0.8 Pa·s have been described depending on extraction conditions and structural features [52,53]. As shear rate increased, viscosity progressively decreased and approached a quasi-Newtonian plateau. This transition occurred at higher shear rates for max GalA (≈80–100 s^−1^) than for max yield and max DE (≈10–20 s^−1^), showing a broader shear rate range over which flow resistance was maintained.

At 200 s^−1^, max GalA held an apparent viscosity of ~0.15 Pa·s, which was 7.5 times higher than max yield (~0.02 Pa·s) and 3.8-fold higher than max DE (~0.04 Pa·s). Comparable viscosity levels at high shear rates have been reported for 2% pectin solutions (0.02 to 0.20 Pa·s), depending on polymer integrity and compositional features [48,54]. The higher viscosity and delayed approach to shear-independent behavior observed for max GalA are consistent with the literature describing increased flow resistance in pectin enriched in GalA and exhibiting reduced structural disruption during extraction [33,53].

To further characterize flow behavior, steady-shear data were fitted to the Power Law model (Table 6). All samples exhibited pseudoplastic behavior, with flow behavior index values below 1 (n = 0.58–0.75). The GalA-optimized pectin displayed the highest consistency index (K = 0.544 Pa·s^n^), suggesting greater resistance to flow, whereas max yield and max DE showed lower consistency values (0.141 and 0.120 Pa·s^n^, respectively). These results agree with the viscosity profiles and suggest that preservation of GalA-rich structure may contribute to enhanced intermolecular interactions in solution.

Overall, the apparent viscosity results show that optimization toward high GalA content yields pectin solutions with greater resistance to flow and reduced shear sensitivity compared with those optimized for yield or DE, reinforcing the functional advantages of GalA-focused extraction strategies.

### 3.15. Solubility

The solubility of the three pectin samples varied with the extraction objective. Max GalA exhibited the highest solubility, (95.2 ± 1.4%), whereas max DE and max yield displayed intermediate solubilities (75.6 ± 2.4% and 74.6 ± 2.2%, respectively). Similar trends have been reported for citrus pectin with high GalA content, where solubility values above 90% were attributed to improved chain hydration and reduced aggregation [55]. In contrast, pectin obtained under yield- or DE-focused conditions often exhibit lower solubility due to partial chain aggregation or structural rearrangements during extraction [22,32].

Compared with citric-acid-extracted orange pectin reported by [22], who obtained solubility values in the range of 70–85% depending on extraction severity and DE, the max yield and max DE samples fall within the reported literature values, while max GalA exceeds the upper range. This difference suggests that optimization toward GalA preservation favors solubilization beyond what is typically achieved through yield-driven extraction alone.

### 3.16. Emulsifying Properties

Emulsifying capacity (EC) differed significantly among samples (*p* < 0.001). As shown in Table 7, pectin at max DE showed the highest EC (14.83 m^2^ g^−1^), followed by max yield (11.81 m^2^ g^−1^) and max GalA (7.56 m^2^ g^−1^). These values reflect the faster interfacial adsorption of more esterified chains, consistent with reports that methyl ester groups improve emulsifying activity in citrus pectin and pectin–protein systems [37]. Ref. [56] likewise reported that systems with greater hydrophobicity improve initial interfacial absorption and thus emulsifying performance.

Emulsion stability (ES) displayed the opposite trend (*p* < 0.001). Max GalA retained the highest turbidity after 10 min (89.70%), while max yield and max DE showed less-stable emulsions (74.91 and 68.5% respectively). The improved stability of max GalA is attributed to its high GalA content, which increases carboxyl group density and may promote more cohesive interfacial films, consistent with stabilization mechanisms proposed for carboxyl-rich pectin systems [37,54].

Overall, the results highlight the contrast between emulsifying activity and stability, where higher esterification enhances initial droplet formation (high EC), while the high GalA extract forms interfacial layers that are more consistent with improved short-term stability (high ES).

### 3.17. Color

Color parameters differed significantly among the powder samples (Table 8). Max GalA showed the highest lightness (L = 82.78) and slightly negative a* values (−1.21), indicating a brighter, more neutral appearance. Max yield and max DE exhibited higher a* and b* values (2.38 and 3.31 for a* and 16.45 and 18.55 for b*, respectively), reflecting a shift toward more yellow tones. These differences are consistent with reports that pectin color is influenced mainly by residual phenolics and minor compositional differences rather than network structure [35,57]. The lower b* value observed for max GalA may reflect reduced retention of yellow pigments under the corresponding extraction conditions. Overall, color served as a supporting indicator of extract purity rather than a functional distinction among samples.

## 4. Conclusions

This study determines that citric acid extraction conditions can be adjusted to selectively maximize pectin yield, GalA content, or DE, resulting in pectin with distinct functional properties. The balance between recovery and functionality was strongly influenced by the combination of extraction pH, temperature, and time. Conditions optimized for yield produced the highest recovery (26.5%) but lower GalA content (67%), reduced viscosity (0.28 Pa·s at 1 s^−1^), weaker gels (122 g hardness), and compact surface morphologies associated with harsher processing conditions. In contrast, optimization toward GalA content yielded pectin with the highest gel hardness (641 g), elevated viscosity across the full shear rate range (0.64 Pa·s at 1 s^−1^), and smoother lamellar surface features, although at substantially lower recovery (5.6%), which may limit industrial applicability unless the functional performance justifies the reduced extraction efficiency. Pectin optimized for DE showed intermediate behavior, combining moderate yield (16.7%), cohesive gel formation, and intermediate rheological properties.

Overall, these results show that maximizing yield does not ensure desirable mechanical or rheological properties, emphasizing the need to select extraction conditions according to the intended application. They also show that GalA content alone is not a reliable predictor of functionality when extraction severity differs.

From a practical perspective, high GalA pectin is better suited for applications requiring strong gelation and high flow resistance, while high yield pectin is more appropriate for less demanding uses. Molecular weight distribution, long-term emulsion stability, and complementary DE determination methods were not evaluated and may provide additional insight into the relationship between severity and functionality. Future work should evaluate emerging and non-thermal pretreatments to improve plant cell wall disruption and pectin release while preserving structural and functional properties, with the aim of increasing recovery without compromising performance.

## Figures and Tables

**Table 1:** Experimental design matrix for the central composite design with experimental and predicted responses of citric acid pectin extraction of orange peel.

Parameters			Experimental			Predicted		
Temp (°C)	Time (min)	pH	Yield (%)	GalA (%)	DE (%)	Yield (%)	GalA (%)	DE (%)
75	70	2.05	9.16	59.92	62.70	10.05	60.58	60.98
75	45	2.05	6.75	77.34	60.28	7.51	72.02	60.16
75	45	2.05	7.84	69.6	61.71	7.51	72.02	60.16
60	60	2.40	4.27	70.92	52.60	3.71	66.21	53.47
60	30	2.40	3.78	70.04	50.88	4.08	69.29	52.50
75	45	2.05	7.59	68.74	60.49	7.51	72.02	60.16
75	45	1.46	18.65	59.42	67.54	19.7	57.29	66.16
87	30	2.40	5.93	68.89	58.71	5.97	69.29	59.01
87	30	1.70	16.06	67.36	62.83	16.02	64.21	62.09
75	45	2.64	3.62	62.69	55.19	3.40	65.86	54.16
75	20	2.05	7.60	65.39	60.24	7.54	65.77	59.35
60	60	1.70	13.05	57.35	63.95	12.54	61.12	65.64
51	45	2.05	4.72	67.46	61.01	5.36	72.02	58.41
60	30	1.70	10.72	61.95	63.62	10.04	64.21	64.67
87	60	2.40	6.25	67.09	60.13	6.36	66.21	59.98
75	45	2.05	7.36	73.96	54.47	7.51	72.02	60.16
96	45	2.05	12.36	69.59	61.28	12.56	72.02	61.69
87	60	1.70	20.28	61.01	61.84	19.28	61.12	63.06
75	45	2.05	8.06	77.16	60.63	7.51	72.02	60.16
75	45	2.05	7.57	71.40	62.00	7.51	72.02	60.16

Experimental design includes six center point replicates (shown individually) at 75 °C, 45 min, and pH 2.05 for pure-error estimation and lack-of-fit testing. GalA: galacturonic acid; DE: degree of esterification.

**Table 2:** Analysis of variance for pectin yield, GalA, and DE, as a function of extraction temperature (°C), time (min), and pH of citric acid extraction of orange peel.

Source	Sum of Squares	DF	Mean Square	F-Value	*p*-Value
**(A) Pectin Yield (%)**					
Model	425.061	9	47.229	85.3	0.001
Temperature	60.474	1	63.249	114.23	0.001
Time	7.313	1	7.112	12.85	0.005
pH	310.878	1	303.646	548.41	0.001
Temperature × Time	0.296	1	0.296	0.54	0.481
Temperature × pH	8.425	1	8.425	15.22	0.003
Time × pH	4.112	1	4.112	7.43	0.021
Temperature^2^	2.717	1	5.176	9.35	0.012
Time^2^	1.435	1	3.001	5.42	0.042
pH^2^	29.41	1	29.41	53.12	0.001
Error	5.537	10	0.554		
Lack of Fit	4.519	5	0.904	4.44	0.064
Pure Error	1.018	5	0.204		
R^2^	0.9871				
Adjusted R^2^	0.9756				
**(B) Galacturonic Acid Content (%)**					
Model	433.49	4	108.37	9.55	0
Time (min)	32.51	1	32.51	2.86	0.111
pH	88.52	1	88.52	7.8	0.014
Time^2^	142.37	1	142.37	12.54	0.003
pH^2^	198.01	1	198.01	17.44	0.001
Error	170.3	15	11.35		
Lack of Fit	101.04	10	10.1	0.73	0.687
Pure Error	69.27	5	13.85		
Total	603.79	19			
R^2^	0.7179				
Adjusted R^2^	0.6427				
**(C) Degree of Esterification (%)**					
Model	246.076	4	61.519	14.92	0
Temp	13.156	1	13.156	3.19	0.094
Time (min)	3.199	1	3.199	0.78	0.392
pH	198.218	1	198.218	48.08	0
Temp × pH	41.585	1	41.585	10.09	0.006
Error	61.846	15	4.123		
Lack of Fit	23.436	10	2.344	0.31	0.948
Pure Error	38.41	5	7.682		
Total	307.922	19			
R^2^	0.7992				
Adjusted R^2^	0.7456				

Sum of squares correspond to sequential (Type I) SS; mean squares and F-tests are based on adjusted (Type III) SS, following Minitab’s default RSM ANOVA.

**Table 3:** Regression equations of the fitted second-order polynomial models for pectin yield, GalA, and DE content in uncoded units of citric acid extraction of orange peel.

Response	Regression Equation
Yield (%)	=46.80 + 0.110·T + 0.146·t − 39.05·pH + 0.00336·T^2^ + 0.00203·t^2^ + 11.62·pH^2^ − 0.2166·T·pH − 0.1366·t·pH
GalA (%)	=−95.50 + 0.0441·T + 1.148·t + 130.10·pH − 0.01390·t^2^ − 29.95·pH^2^
DE (%)	=148.10 − 0.914·T + 0.0323·t − 46.30·pH + 0.481·T·pH

All variables are expressed in actual (uncoded) units and correspond to the final fitted response surface models. T: temperature (°C); t: time (min); GalA: galacturonic acid; DE: degree of esterification.

**Table 4:** Experimental values of yield, GalA, and DE obtained under the citric acid extraction conditions corresponding to the maximum of each individual response.

Response Target	Extraction Conditions	Experimental Results		
		Yield (%)	GalA (%)	DE (%)
Max Yield	96 °C, 70 min, pH 1.46	26.5 ± 1.1	67.1 ± 6.6	63.4 ± 1.4
Max GalA	74 °C, 42 min, pH 2.17	5.6 ± 0.3	84.1 ± 3.6	62.8 ± 1.7
Max DE	50 °C, 70 min, pH 1.46	16.7 ± 0.2	65.0 ± 4.2	67.0 ± 1.0

GalA: galacturonic acid; DE: degree of esterification.

**Table 5:** Textural parameters of pectin obtained under conditions maximizing yield (96 °C, 70 min, pH 1.46), GalA (74 °C, 42 min, pH 2.17), and DE (50 °C, 70 min, pH 1.46).

Parameter	Max Yield	Max GalA	Max DE
Hardness (g)	121.82 ± 16.53 ᶜ	641.30 ± 46.40 ᵃ	336.84 ± 3.01 ᵇ
Adhesiveness (g·s)	17.01 ± 16.12 ᵃ	28.67 ± 16.51 ᵃ	25.55 ± 5.20 ᵃ
Cohesiveness	0.77 ± 0.14 ᵃ	0.79 ± 0.08 ᵃ	0.67 ± 0.05 ᵃ
Springiness	1.00 ± 0.05 ᵃ	1.00 ± 0.01 ᵃ	1.00 ± 0.02 ᵃ
Resilience	0.78 ± 0.06 ᵃ	0.75 ± 0.06 ᵃ	0.81 ± 0.06 ᵃ
Gumminess & Chewiness (g)	93.60 ± 18.08 ᶜ	507.12 ± 67.76 ᵃ	226.90 ± 17.99 ᵇ

Different superscript letters within the same row indicate significant differences between means (Tukey, *p* < 0.05). Because springiness values were approximately unified for all samples, gumminess and chewiness produced numerically similar values and are therefore presented together to avoid redundancy. GalA: galacturonic acid; DE: degree of esterification.

**Table 6:** Power Law model parameters of 2.0% (*w/w*) pectin solutions obtained under optimized extraction conditions.

Sample	n	K (Pa·s^n^)
Max Yield	0.597 ± 0.039 ᵇ	0.135 ± 0.035 ᵇ
Max GalA	0.759 ± 0.003 ᵃ	0.523 ± 0.019 ᵃ
Max DE	0.774 ± 0.027 ᵃ	0.118 ± 0.006 ᵇ

K, consistency index; n, flow behavior index. Values are mean ± SD (n = 3) from Power Law fits over 1–200 s^−1^. Different letters indicate significant differences within columns by Tukey’s HSD test (*p* < 0.05). R^2^: max yield = 0.856 ± 0.041; max GalA = 0.993 ± 0.000; and max DE = 0.934 ± 0.003. GalA: galacturonic acid; DE: degree of esterification.

**Table 7:** Emulsifying capacity and emulsion stability of pectin extracts.

Sample	EC (m^2^ g^−1^)	ES (%)
Max Yield	11.81 ± 0.66 ᵇ	74.91 ± 3.97 ᵇ
Max GalA	7.56 ± 0.11 ᶜ	89.70 ± 1.56 ᵃ
Max DE	14.83 ± 0.23 ᵃ	68.50 ± 4.14 ᵇ

Values are expressed as mean ± standard deviation (n = 3). Different superscript letters within a column indicate significant differences among samples (Tukey, *p* < 0.05). EC = emulsifying capacity; ES = emulsion stability. GalA: galacturonic acid; DE: degree of esterification.

**Table 8:** Color parameters of pectin samples obtained under optimized extraction conditions (mean ± SD, n = 3).

Sample	L* (Lightness)	a* (Red/Green)	b* (Yellow/Blue)
Max Yield	78.20 ± 1.12 ^b^	2.38 ± 0.08 ^b^	16.45 ± 0.73 ^b^
Max GalA	82.68 ± 0.17 ^a^	−1.21 ± 0.01 ^a^	6.36 ± 0.16 ^a^
Max DE	76.26 ± 1.85 ᶜ	3.31 ± 0.31 ᶜ	18.55 ± 0.18 ᶜ

Values are means ± SD; n = 3. Different superscript letters within a column indicate significant differences among samples, Tukey *p* < 0.05. GalA: galacturonic acid; DE: degree of esterification.

## Data Availability

Data are contained within the article.
